# Simultaneity and Temporal Order Judgments Are Coded Differently and Change With Age: An Event-Related Potential Study

**DOI:** 10.3389/fnint.2018.00015

**Published:** 2018-04-26

**Authors:** Aysha Basharat, Meaghan S. Adams, William R. Staines, Michael Barnett-Cowan

**Affiliations:** Department of Kinesiology, University of Waterloo, Waterloo, ON, Canada

**Keywords:** aging, audiovisual, event-related potentials, multisensory integration, simultaneity perception, temporal order perception, temporal binding window

## Abstract

Multisensory integration is required for a number of daily living tasks where the inability to accurately identify simultaneity and temporality of multisensory events results in errors in judgment leading to poor decision-making and dangerous behavior. Previously, our lab discovered that older adults exhibited impaired timing of audiovisual events, particularly when making temporal order judgments (TOJs). Simultaneity judgments (SJs), however, were preserved across the lifespan. Here, we investigate the difference between the TOJ and SJ tasks in younger and older adults to assess neural processing differences between these two tasks and across the lifespan. Event-related potentials (ERPs) were studied to determine between-task and between-age differences. Results revealed task specific differences in perceiving simultaneity and temporal order, suggesting that each task may be subserved via different neural mechanisms. Here, auditory N1 and visual P1 ERP amplitudes confirmed that unisensory processing of audiovisual stimuli did not differ between the two tasks within both younger and older groups, indicating that performance differences between tasks arise either from multisensory integration or higher-level decision-making. Compared to younger adults, older adults showed a sustained higher auditory N1 ERP amplitude response across SOAs, suggestive of broader response properties from an extended temporal binding window. Our work provides compelling evidence that different neural mechanisms subserve the SJ and TOJ tasks and that simultaneity and temporal order perception are coded differently and change with age.

## Introduction

The ability to integrate information from various sensory modalities is imperative for the optimal perception of external events. In order for multisensory integration to occur, the central nervous system (CNS) must integrate temporally and spatially different signals from various sensory organs. These stimuli must fall in a specific range of temporal offsets termed the temporal binding window (TBW) (Meredith et al., 1987; Stein et al., 1988). The TBW appears to be quite malleable and has been shown to change throughout the lifespan (Powers et al., 2009; Hillock et al., 2011; Hillock-Dunn and Wallace, 2012; de Boer-Schellekens and Vroomen, 2014; Bedard and Barnett-Cowan, 2016) as well as following injury such as concussion (Wise and Barnett-Cowan, 2018). It tends to be wider during early childhood and becomes fine-tuned during middle childhood around 8–10 years of age (Lewkowicz, 1996; Hillock et al., 2011; Hillock-Dunn and Wallace, 2012), a critical time for changes in the development of multisensory integration as found in other studies (Gori et al., 2008). At the opposite end of the developmental spectrum, normally aging older adults show a wider TBW indicating that during later stages of life it becomes increasingly difficult to discriminate the temporal order of events (Poliakoff et al., 2005; Ulbrich et al., 2009; Setti et al., 2011a,b; Chan et al., 2014a,b; Diederich and Colonius, 2015; Bedard and Barnett-Cowan, 2016). This increase in the width of the TBW is potentially of concern as information from separate events that should be encoded as temporally separate are more likely to be integrated as one event which could lead to decision making and motor errors.

The importance of integrating multisensory information in time is exemplified by studies showing that erroneous integration of multisensory information is associated with increased fall risk (Setti et al., 2011a,b; Mahoney et al., 2014) and decreased speech comprehension (Maguinness et al., 2011; Setti et al., 2013). Importantly, these deficits cannot only be attributable to changes in unisensory processing (Setti et al., 2011b). Furthermore, although older adults show reduced visual contrast sensitivity (Derefeldt et al., 1979) and sound detection thresholds, specifically at higher frequencies (Gordon-Salant, 2005), they can benefit from multisensory integration. For example, it has been found that older adults benefit from integrating bimodal cues compared to unimodal cues more than younger adults (Laurienti et al., 2006; Diederich et al., 2008; Freiherr et al., 2013). Due to the plasticity of the TBW, recalibration to decrease TBW size can be thought of as a rehabilitative technique that can be used to prevent decision-making errors and increase motor control (Powers et al., 2009; Chan et al., 2014a,b). In order to assess change in TBW size, however, it is important to establish how to measure the size of the TBW.

Various psychophysical measures and illusions that make use of temporally disparate signals are utilized to determine the size of the TBW (Sekuler et al., 1997; Shams et al., 2000; Zampini et al., 2005; van Eijk et al., 2008; Love et al., 2013). These methods involve varying the stimulus onset asynchrony (SOA) between the two stimuli in order to determine the point of subjective simultaneity (PSS) and the TBW. Simultaneity judgment (SJ) and temporal order judgment (TOJ) tasks have often been utilized to extract the PSS and the TBW. In the SJ tasks, participants are subjected to two stimuli of differing modalities and are asked to determine whether the two stimuli are simultaneous, while in the TOJ task, participants are asked to determine which stimulus came first (van Eijk et al., 2008). Although the stimuli for these two tasks are identical in nature, SJ and TOJ have previously been shown to be supported by different perceptual mechanisms and are not representative of the same perceptual process as evidenced by differing estimates of perceptual latency between tasks (Mitrani et al., 1986; Vatakis et al., 2008; Barnett-Cowan and Harris, 2009; Barnett-Cowan and Harris, 2011; Love et al., 2013). In sum, there are multiple means to assess TBW size, and therefore it is thought that differences among these tasks may relate to different underlying neural mechanisms (Linares and Holcombe, 2014), or that higher-level decision-making contributes to the differences that exist between the two tasks. Previously, our lab showed that with aging, the ability to discriminate temporal order of multisensory events is diminished, while there appears to be no change in the ability to discriminate simultaneity (Bedard and Barnett-Cowan, 2016). In the present paper, we use electroencephalography (EEG) to characterize temporal changes in cortical excitability associated with SJ and TOJ tasks in younger and older adults.

What do we know already from neural imaging studies regarding the processing of multisensory information in time? There has been some research conducted using imaging techniques to investigate the mechanisms underlying the SJ and TOJ tasks; however, they have not directly compared performance on the two tasks in younger and older adults. Adhikari et al. (2013) used a TOJ task with fMRI and found that judgment of temporal order involved network activity between the parietal and prefrontal cortices in younger adults. They compared activation maps of synchrony versus asynchrony and found that there was activation in the right superior temporal gyrus, inferior parietal lobe, supramarginal gyrus, left medial frontal gyrus, and right middle frontal gyrus. They therefore concluded that the left temporal and parietal cortices and the right frontal cortex are involved in synchrony perception. Additionally, Dhamala et al. (2007) asked younger adults to judge whether audiovisual stimuli presented were simultaneous, whether a sound was presented first, a light was presented first, or if they could not determine temporal order using an fMRI design. The results from the study indicated that the primary auditory and visual sensory cortices, parietal, and prefrontal cortices were activated during perception of asynchrony. On the other hand, the perception of synchrony recruited the superior colliculus and disengaged the inferior parietal lobule. Setti et al. (2011a) compared temporal order perception in younger and older adults using event-related potentials (ERPs) and determined that older participants had a smaller visual P1 amplitude than younger participants in the TOJ task. Here, planned comparisons indicated that older adults had a significantly smaller P1 amplitude at the SOA of 270 ms but not at the SOA of 70 ms, indicating that this difference in amplitude is not likely attributable to the extended TBW in older adults. In addition, there were no main effects of age found for the auditory N1 ERPs, however, planned comparisons revealed again that older adults had significantly smaller N1 ERPs for the SOA of 270 ms, but not the SOA of 70 ms. Topographical maps showed that the overall activity in occipital and frontal regions was more distributed in older compared to younger participants. Setti et al. (2011a) results further indicate that determining the temporal order of events is difficult for older adults when audiovisual stimuli are separated by large delays. Taken together, the imaging literature, in agreement with behavioral literature, indicates that different neural networks may be involved in simultaneity versus temporal order perception and that EEG may be a useful tool in determining such differences between younger and older adult neural activity.

The purpose of this study is to characterize the behavioral (TBW, PSS) as well as electrophysiological differences between SJ and TOJ tasks. It is hypothesized that (i) the PSS between the two tasks will not be correlated for both younger and older adults (van Eijk et al., 2008; Love et al., 2013; Bedard and Barnett-Cowan, 2016). It is also hypothesized that (ii) the mean PSS and the mean TBW will be significantly different between SJ and TOJ tasks for both younger and older adults (Love et al., 2013). Furthermore, it is hypothesized that (iii) a wider TBW will be observed in older adults for the TOJ task, but not for the SJ task (Bedard and Barnett-Cowan, 2016). Additionally, we aimed to assess the reported differences that exist between younger and older adults in SJ and TOJ perception. Visual P1 and auditory N1 ERP components were analyzed as they have been found to differ between younger and older adults (De Sanctis et al., 2008; Setti et al., 2011a). Additionally, they have previously been linked with perceptual processing (Giard and Peronnet, 1999; Molholm et al., 2002; De Sanctis et al., 2008; Setti et al., 2011a) and modulation of perception through attention (Hillyard et al., 1998; Setti et al., 2011a). Therefore, it is hypothesized that (iv) within each group, there will be no significant differences in visual P1 and auditory N1 amplitude and latency components between the two tasks for the control conditions (processing of unimodal stimuli), as they are representative of unisensory integration. It is also hypothesized that (v) older adults will have smaller P1 amplitudes compared to younger adults for the TOJ task in the control condition (Gazzaley et al., 2008; Setti et al., 2011a). Finally, it is hypothesized that (vi) there will be no main effect of age on the auditory N1 amplitude and latency for the experimental conditions (processing of multisensory stimuli) for the TOJ task (Setti et al., 2011a). Overall our study is designed to determine whether both cortical and behavioral responses elicited by audiovisual simultaneity perception and temporal order discrimination differ among younger and older adults. To our knowledge, this is the first study that compares the neural correlates of younger and older adults obtained from the SJ and TOJ tasks.

## Materials and Methods

### Participants

Participants (*n* = 56) were recruited from the University of Waterloo as well as through the Waterloo Research in Aging Participant Pool (WRAP). The WRAP program ensures all participants are healthy older adults over the age of 60 with no significant neurological impairments (i.e., Alzheimer’s disease, Parkinson’s disease, stroke, epilepsy, etc.).

Participants between the ages of 19 and 79 years of age were included in this study. Prior to participation, the subjects completed a clinical information form where they indicated (yes/no) if they had normal or corrected-to-normal vision, if they had normal or corrected-to-normal hearing, and if they believed they would be comfortable with the equipment and procedures of this study. If participants answered no to any of the above questions, they were subsequently excluded from the study.

A sample size of 28 participants for each group (young and old) was selected following an initial collection of 18 participants for each group to ensure sufficient power to determine differences between the two tasks and differences between the two cohorts. The older adult group had a mean age of 68.95 (*SD* = 0.895, 17 males), while the young adult group had a mean age of 21.6 (*SD* = 0.365,13 males). In appreciation of their participation, participants received a $10 per hour remuneration. This study was approved by and carried out in accordance with the recommendations of the University of Waterloo’s Human Research Ethics Committee with written informed consent from all participants.

### Experimental Set-up

Each participant completed two experimental tasks while seated in front of a 23.6 inch ViewSonic V3D245 computer monitor (resolution 1920 × 1080, 120 Hz) in a sound-proof booth with their head stabilized by a chin rest. Visual stimuli were presented on the monitor at a viewing distance of 57 cm, in the form of white circles. Auditory stimuli were emitted from two speakers (Altec Lansing Multimedia computer speaker system, ACS95W) adjacent to the monitor such that they were 66 cm apart and because they played sound at equal intensity the midpoint of the distance between the speakers coincided with the visual stimulus. A MacBook Pro (OS 10.9 Mavericks) that resided outside of the booth was used to run the tasks. VPixx Technologies ProPixx hardware and DataPixx software version 3.01 was utilized for this experimental procedure to ensure that audio and visual stimuli were accurately presented relative to each other in real time with <1 ms accuracy. The RESPONSEPixx handheld five-button response box was utilized by participants to input their responses to each trial. This response box consisted of two illuminated buttons, where participants recorded their responses by pressing the corresponding buttons, as instructed for each task. Participants were told to respond as accurately as possible as opposed to giving a speeded response. All participants received the same instructions for each task.

### Tasks

Participants completed the SJ and TOJ tasks in a randomized order. For both tasks, a central fixation cross (visual angle = 0.5°) was presented on the screen, and participants were instructed to fixate on this cross throughout the experimental procedure. Participants were presented practice trials prior to commencement of each of the experimental tasks. After the practice trials, the researcher then left the booth and closed the door. The researcher started the trial and monitored participant progress on a laptop from outside the booth.

**FIGURE 1:**
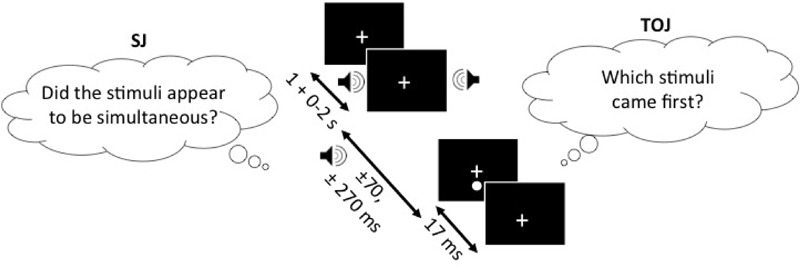
SJ task (left) and the TOJ task (right), presented with the SOAs of ±70 and ±270 ms. In both of the tasks, the one stimulus of the audiovisual pair (sound in this example) can appear 1–3 s following the fixation cross and another stimulus (light in this example) appears either ±70 or ±270 ms relative to the other stimulus. The figure depicts the auditory stimulus (i.e., beep) as presented 270 ms before the visual stimulus (i.e., flash). Note, that the design is the same for both of the tasks and only the question asked is different.

In the SJ task, participants were to report, using the response buttons, whether they perceived the auditory and visual stimuli to be occurring simultaneously (right button) or not (left button). Visual stimuli in the form of a 0.4° white circle (49.3 cd/m^2^) against a black background (0.3 cd/m^2^), which appeared 2° below the fixation cross for 17 ms, were either preceded or followed by an auditory beep (1850 Hz, 7 ms, 71.7 dB) at SOAs of ±70 ms and ±270 ms (-ve = sound first). Testing was completed in one session which consisted of five trial blocks with 25 repetitions of each SOA per block, for a total of 125 trials per SOA and 500 trials in total for each task. Participants were given short breaks between blocks in order to decrease boredom or inattentiveness during each block. The experimental design of the TOJ task was identical to the SJ task with the exception of the question that participants were asked to respond to. In the SJ task, participants were asked to determine whether or not the light and the sound were simultaneous whereas in the TOJ task, participants were asked to determine whether or not light (right button) appeared before sound (left button) (**Figure 1**).

### Electroencephalography

Participants performed the two tasks while electroencephalogram (EEG) was acquired from a 32-channel electrode cap (Quick-Cap, Neuroscan) that was positioned using the 10/20 international system guidelines. All EEG channels were referenced to linked mastoids. EEG data was digitized at 1000 Hz (Neuroscan 4.5, SynAmps2, Compumedics, NC, United States) and channel impedances were maintained at <5 KΩ. Epochs of length 1,000 ms were extracted starting 200 ms prior to the onset of the first stimulus in a sequence where two stimuli were presented. The epochs were bandpass filtered from 1 Hz to 30 Hz (24 dB/octave), and baseline corrected to the pre-stimulus interval (-200 ms - 0 ms). Epochs were extracted on a trial-by-trial basis by the researcher in order to ensure that all trials with artifacts were removed. Epochs were rejected based on the criteria of ±100 μV and a visual inspection to remove epochs corrupted with eye-blinks and muscle movement. Additionally, epochs with excessive alpha activity were also rejected. As the participants were seated in a dark, soundproof booth, it was not possible for the researchers to ensure that the participants were attentive, thus an increase in alpha activity was used as a monitoring technique to remove the trials during which the participants may not have been attending or focusing on the fixation cross.

For each task, individual average ERPs were calculated for each participant and then a group average was calculated. ERP peak amplitudes and latency values for the visual P1 waveform were extracted from occipital and parietal electrodes (O2 and P4) as maximal peaks were found at these sites. Auditory N1 ERP peak amplitudes and latencies were extracted from the fronto-central electrode (FCz). Note that although only O2 and P4 electrodes and the FCz electrode were utilized for the extraction of the visual P1 and auditory N1 ERPs, respectively, the decision to use these electrodes was based on the work conducted by Giard and Peronnet (1999); Molholm et al. (2002), and Setti et al. (2011a). Furthermore, a qualitative analysis of the data was conducted in order to verify that the electrodes with the largest effect were chosen for analysis. During the qualitative analysis, it was found that the P4 electrode showed larger effects than the O2 electrode for some of the conditions and hence it was also utilized in the analysis for the visual P1 ERP component. For each condition, individual average ERPs were created from the epochs. Analysis parameter intervals were chosen based on previous research (Giard and Peronnet, 1999; Molholm et al., 2002; Setti et al., 2011a) as well as a visual investigation of individual averages, and grand-average ERP waveforms. The parameter intervals were extracted for each SOA and categorized as either control or experimental conditions. Visual P1 and auditory N1 amplitudes were extracted by obtaining the maximum peak in the parameter extraction window (defined below). The control conditions were characterized as representative of unisensory responses where amplitude and latency values were extracted from the first stimuli in each trial (i.e., control visual P1 ERP extracted from the response to ‘flash’ in flash-beep trials). The experimental conditions on the other hand, were categorized as representative of multisensory integration where amplitude and latency values were extracted from the second stimuli in each trial (i.e., experimental visual P1 ERP extracted from the response to ‘flash’ in beep-flash trials).

For the control conditions, the ERPs were extracted after the presentation of the first stimulus. For both the visual P1 and auditory N1 ERP control conditions, an extraction parameter of 80–180 ms was utilized. As for the experimental condition, the extraction parameter was chosen after the second stimulus was presented. For SOA of -270 ms (-ve = sound first), time-locked to light, experimental visual P1 ERPs were extracted from the 80 to 180 ms interval. For the SOA of -270 ms, time-locked to sound, experimental visual P1 ERPs were computed for the 350–440 ms interval. For the SOA of -70 ms, time-locked to light, experimental visual P1 ERPs were extracted from 80 to 170 ms interval. For the SOA of -70 ms, time-locked to sound, experimental visual P1 ERPs were computed for the 150–240 ms interval. For the SOA of 70 ms, time-locked to light, experimental auditory N1 ERPs were extracted from 170 to 220 ms interval. For the SOA of 70, time-locked to sound, experimental auditory N1 ERPs were extracted from 85 to 150 ms interval. For the SOA of 270 ms, time-locked to light, experimental auditory N1 ERPs were extracted from 350 to 450 ms interval. For the SOA of 270 ms, time-locked to sound, experimental auditory N1 ERPs were extracted from 85 to 150 ms interval.

### Statistical Analysis

#### Behavioral Analysis

To estimate the PSS values and the certainty with which participants made their judgments (the TBW) for each task, psychometric functions were fitted to each participant’s responses as a function of SOA using SigmaPlot version 12.0. Each task was analyzed individually for each participant, with participant data fitted by either a Gaussian function (for the SJ task; Eq. 1) or a sigmoidal function (for the TOJ task; Eq. 2):

(1) $$y=a.e^{\left(-0.5{\left(\frac{x-xø}{b}\right)}^{2}\right)}$$

Where *a* is the amplitude, *xø* is the PSS, and *b* is the standard deviation.

(2) $$y=\frac{100}{1+e^{\frac{x-xø}{b}}}\%$$

Where *xø* is the PSS, and *b* is the standard deviation (slope between 0.25 and 0.75)

The best parameters were found for each participant separately, and those participants who chose the same response for an entire block of 100 trials for any task (i.e., chose “simultaneous” for one entire block of SJ) were excluded from further analysis. As we are interested in the relationships between TBWs obtained from different tasks and not their absolute size, we chose to analyze the *b* values of these psychometric functions as proxy for the size of the TBW to avoid discrepancies in the literature that differ when defining the absolute size of the TBW.

**FIGURE 2:**
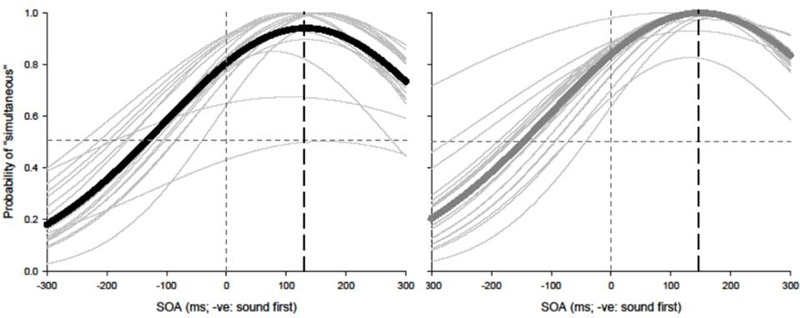
Simultaneity judgment (SJ): average (thick lines) and individual (thin lines). Gaussian data fits showing that younger adults (black) and older adults (gray) require the visual stimulus to be presented approximately 126 and 147 ms prior to auditory stimulus in order to perceive the two stimuli as simultaneous, respectively.

**FIGURE 3:**
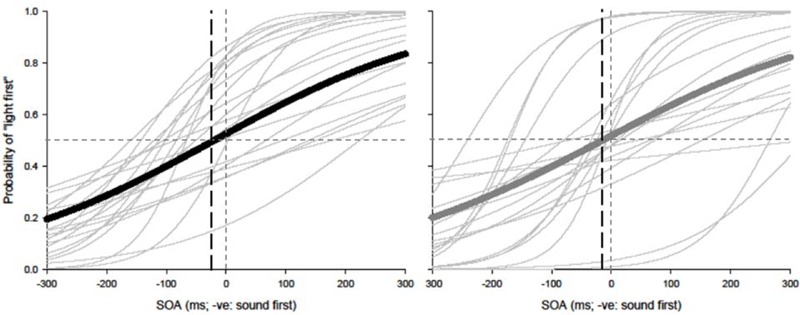
Temporal order judgment (TOJ): average (thick lines) and individual (thin lines). Sigmoidal data fits showing that younger adults (black) and older adults (gray) require the auditory stimulus to be presented approximately 20 and 7 ms prior to visual stimulus in order to perceive the two stimuli as simultaneous, respectively.

Paired *t*-tests were conducted to test for equality of variance for PSS and TBWs within each group. Using a within-subjects design, correlations were assessed between tasks for all participants while controlling for age. Correlations were found utilizing the Pearson correlation coefficient with α set at 0.05. The best parameter fits were found for each participant separately using Sigmaplot 12.0 and PSSs and TBWs were extracted for SJs and TOJs individually. Average psychometric functions for younger and older adults constructed using Sigmaplot 12.0 are represented in **Figure 2** (SJ *R*^2^ *M*: 0.928; Median: 0.930; *SD*: 0.046; *SE*: 0.006) and **Figure 3** (TOJ *R*^2^ *M*: 0.849; Median: 0.912; *SD*: 0.163; *SE*: 0.024).

#### Event-Related Potential Analysis

In order to test whether or not the amplitude and latency values differed between each task at each of the SOAs for each participant, the ‘control’ and ‘experimental’ conditions were analyzed using repeated measures ANOVAs with a 2 (task) × 2 (SOA) design. This analysis was utilized for the auditory N1 component amplitude and latency differences for both visual and auditory time-locked conditions within each group. The same analysis was utilized to test for the visual P1 component differences for both auditory and visual time-locked components within each group. In addition to within subject comparisons, the results obtained from younger and older adults were compared using RM ANOVAs for each condition for both of the tasks. RM ANOVAs with a 2 (group) × 2 (task) × 2 (SOA) design for the control and experimental conditions were conducted for both auditory and visual time-locked components. Statistical analyses were conducted using SPSS (v21).

Participants were excluded from further analysis either if they responded 100% for one category (e.g., 100% “simultaneous”) or if their parameters were poorly estimated (*r*^2^ ≤ 0.2). One younger and three older adults were excluded from further analysis due to poor parameter estimates (*r*^2^ < 0.2). Five younger participants and four older participants were excluded from further analysis due to 45 or more trials out of 125 showing high alpha activity (frequency range of 7.5–12.5 Hz), blinks, or excessive muscle movement. Thus in total, 22 younger and 21 older adults were included in the final analysis.

## Results

### Behavioral Results

**FIGURE 4:**
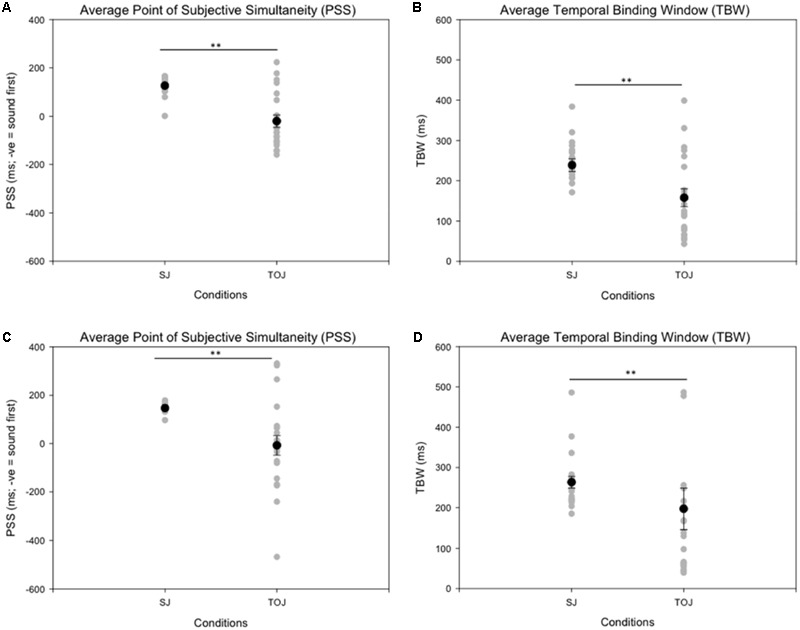
Average PSS (left) and TBW (right) for the SJ and TOJ tasks. The gray circles represent each individual participant’s data whereas the black circle represents the average obtained from younger adults **(A,B)** and older adults **(C,D)**. Significant differences in PSS as well as TBW were obtained between the two tasks. ^∗∗^*p* < 0.01. Error bars are ±1 SEM.

**FIGURE 5:**
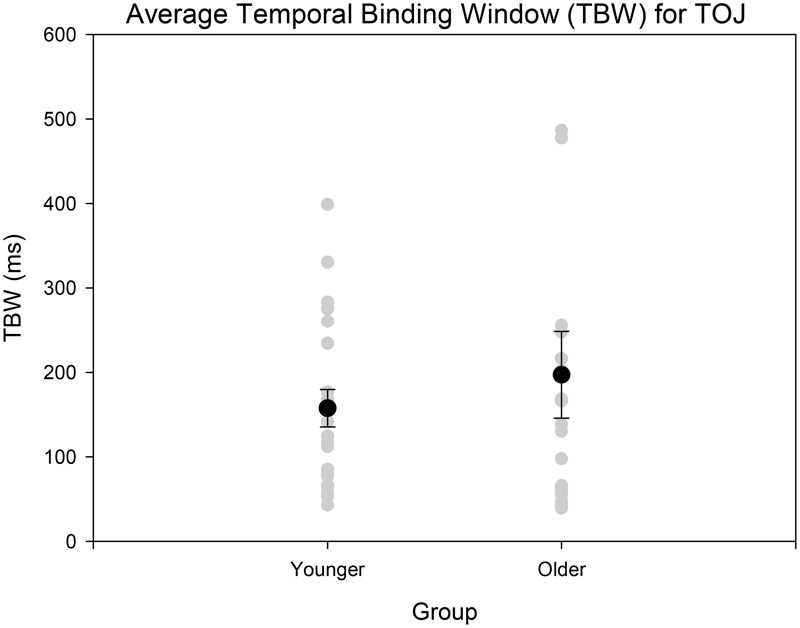
Average TBW for younger and older adults for the TOJ task. The gray circles represent each individual participant’s data whereas the black circle represents the average obtained from each group. A significant difference between the means of the two groups was not obtained. Error bars are ±1 SEM.

Age-controlled correlations were conducted for all participants for the PSS. Planned paired *t*-tests were conducted to determine whether the mean PSSs and TBWs would be significantly different from the two tasks within younger and older adults. Within the younger group, the paired *t*-tests revealed a significant difference in the SJ PSS values (*M* = 126.40, *SD* = 36.14) and TOJ PSS values (*M* = -20.45, *SD* = 115.98); [*t*(20) = 5.24, *p* < 0.001; **Figure 4**]. Furthermore, the *t*-tests also revealed a significant difference between the SJ TBW (*M* = 238.48, *SD* = 72.05) and TOJ TBW values (*M* = 157.64, *SD* = 102.03); [*t*(20) = 4.96, *p* < 0.001; **Figure 4**]. Within the older group, paired *t*-tests revealed a significant difference in the SJ PSS values (*M* = 146.65, *SD* = 17.10) and TOJ PSS values (*M* = -7.45, *SD* = 185.66); [*t*(19) = 3.50; *p* = 0.002; **Figure 4**]. The paired *t*-tests also revealed a significant difference between the SJ TBW (*M* = 257.37, *SD* = 63.62) and TOJ TBW (*M* = 154.14, *SD* = 132.60); [*t*(20) = 3.74, *p* = 0.001; **Figure 4**]. Pearson’s correlations were also conducted, and no significant Pearson’s correlations were found for PSS between SJ and TOJ within both younger [*r*(21) = -0.206, *p* = 0.371] or older adults [*r*(21) = -0.135, *p* = 0.56]. Planned independent *t*-tests were conducted to determine age-related significant differences between the two tasks. Although older adults had wider TBWs compared to younger adults for both the SJ and the TOJ task, the independent *t*-test revealed no significant difference between the older TBW (*M* = 197.09, *SD* = 235.47) and younger TBW (*M* = 157.64, *SD* = 102.03) for the TOJ task; [*t*(40*)* = 0.704, *p* = 0.485; **Figure 5**]. This was also the case for the SJ task where the older TBW (*M* = 263.06, *SD* = 67.27) and the younger TBW (*M* = 238.48, *SD* = 72.05) were not significantly different [*t*(40) = 1.143, *p* = 0.260; **Figure 5**].

### Event-Related Potential Results

#### Within Subjects Results

**FIGURE 6:**
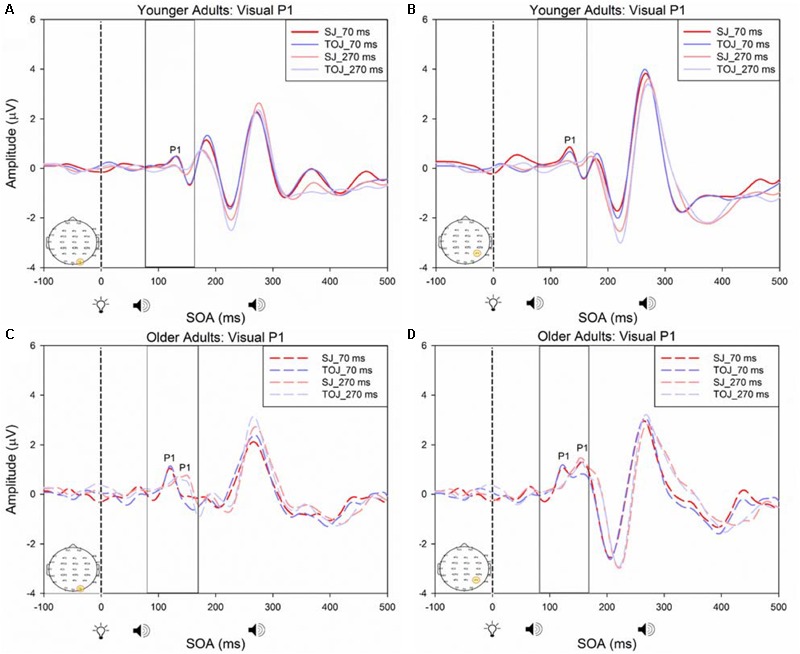
Average amplitude (μV) of visual P1 ERPs from the within-subjects analysis obtained from the control condition from younger **(A,B)** and older **(C,D)** adults in response, and time-locked, to the flash stimulus (light bulb icon) from the visual-auditory conditions (sound, speaker icons, presented 70 or 270 ms after light) obtained from the O2 and P4 electrodes for the TOJ and SJ tasks.

**FIGURE 7:**
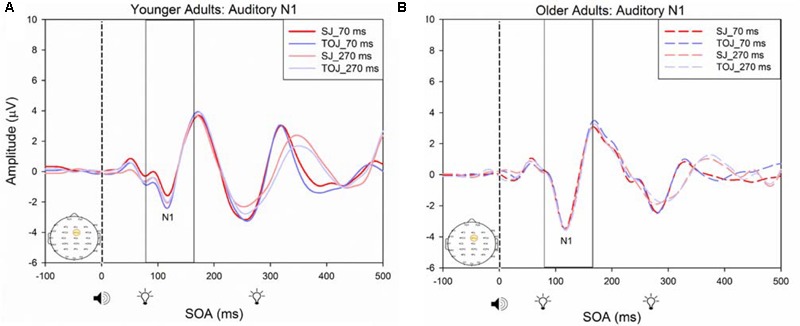
Average amplitude (μV) of auditory N1 ERPs from the control condition from younger **(A)** and older **(B)** adults in response, and time-locked, to the beep stimulus (sound icon) from the auditory-visual conditions (light, light bulb icon, presented 70 or 270 ms after sound) obtained from the FCz electrode for the TOJ and SJ tasks.

A 2 (SOA: 70 ms, 270 ms, time-locked to light) × 2 (task: SJ, TOJ) ANOVA design was utilized to extract the amplitudes and latencies for the control visual P1 condition. A 2 (SOA: -270 ms, -70 ms, time-locked to sound) × 2 (task: SJ, TOJ) RM ANOVA design was utilized to extract the amplitudes and latencies for the control auditory N1 ERPs. As predicted, no main effect of task was found for the visual P1 amplitude [*F*(1,20) = 1.607, *p* = 0.220] and latency [*F*(1,20) = 1.884, *p* = 0.185] from the O2 electrode (**Figure 6**) and the visual P1 amplitude [*F*(1,20) = 0.641, *p* = 0.433] and latency [*F*(1,20) = 1.642, *p* = 0.215] from the P4 electrode (**Figure 6**) as well as the auditory N1 amplitude [*F*(1,21) = 0.999, *p* = 0.329] and latency [*F*(1,21) = 0.487, *p* = 0.493] from the FCz electrode in younger adults (**Figure 7**). This was also the case in older adults, as there was no main effect of task for the visual P1 amplitude [*F*(1,19) = 3.52, *p* = 0.560] and latency [*F*(1,20) = 0.905, *p* = 0.353] from the O2 electrode (**Figure 6**) and the visual P1 amplitude [*F*(1,20) = 0.345, *p* = 0.563] and latency [*F*(1,20) = 1.136, *p* = 0.299] from the P4 electrode (**Figure 6**). Additionally, there was also no main effect of task for the auditory N1 amplitude [*F*(1,20) = 1.170, *p* = 0.292] and latency [*F*(1,20) = 3.754, *p* = 0.067] (**Figure 7**) in the older group. Although there was no main effect of task, the results did indicate an interaction between SOA and task for the visual P1 peak latency obtained from the O2 electrode, when the SOAs of 270 ms and 70 ms were time-locked to light; [*F*(1,20) = 4.74, *p* = 0.042] in the older adult group.

#### Between Subjects Results

**FIGURE 8:**
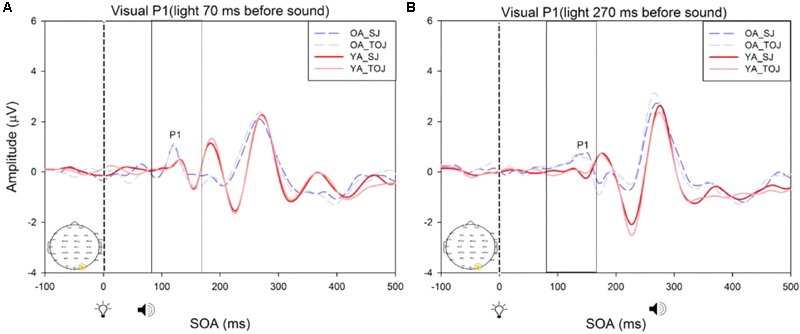
Average amplitude (μV) of visual P1 ERPs from the between-subjects analysis obtained from the control condition from younger and older adults in response, and time-locked, to the flash stimulus (light bulb icon) from the visual-auditory conditions [sound, speaker icons, presented 70 ms after light **(A)** and 270 ms after light **(B)**] obtained from the O2 electrode for the TOJ and SJ tasks.

A 2 (group: older and younger) × 2 (task: SJ and TOJ) × 2 (SOA: 70 ms, 270 ms; time-locked to light) design was used to test the control visual P1 amplitude and latency differences between older and younger adults. A 2 (SOA: -270ms, -70 ms, time-locked to sound) × 2 (task – SJ, TOJ) RM ANOVA design was utilized to extract the amplitudes and latencies for the control auditory N1 condition. A main effect of age was found for the visual P1 latency from the O2 electrode [*F*(1,19) = 14.39, *p* = 0.001], where older adults showed a later latency (*M* = 126.05, *SD* = 2.98) compared to younger adults (*M* = 117.35, *SD* = 2.174) (**Figure 8**). No main effect of age was found for the visual P1 amplitude from both the O2 electrode [*F*(1,18) = 2.155, *p* = 0.159] and the P4 electrode [*F*(1,20) = 3.046, *p* = 0.096]. Additionally, no main effect of task was found for the visual P1 amplitude [*F*(1,18) = 0.043, *p* = 0.839] and latency [*F*(1,17) = 0.083, *p* = 0.777] from both the O2 electrode. This was also the case for the amplitude [*F*(1,20) = 0.936, *p* = 0.345] and latency [*F*(1,20) = 3.065, *p* = 0.095] obtained from the P4 electrode. Furthermore, a main effect of SOA was found for the visual P1 amplitude [*F*(1,20) = 9.70, *p* = 0.005] and latency [*F*(1,20) = 5.27, *p* = 0.033] from the P4 electrode. See Supplementary Figure 1 for boxplots of peak amplitudes and latencies from each participant. No main effect of age was found for the auditory N1 amplitude [*F*(1,20) = 0.994, *p* = 0.331] and latency [*F*(1,20) = 3.174, *p* = 0.09] from the FCz electrode. Additionally, no main effect of task was found for the auditory N1 amplitude [*F*(1,20) = 0.999, *p* = 0.330] and latency [*F*(1,20) = 3.96, *p* = 0.06]. No main effect of SOA was found for the auditory N1 amplitude [*F*(1,20) = 1.00, *p* = 0.329] and latency [*F*(1,20) = 0.242, *p* = 0.628] (Supplementary Figure 2).

#### Experimental Condition

**FIGURE 9:**
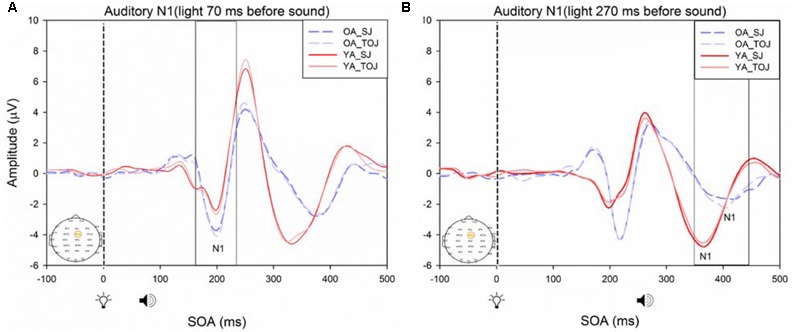
Average amplitude (μV) of auditory N1 ERPs from the experimental condition from younger and older adults time-locked to light in response to the beep stimulus (sound icon) from the visual-auditory conditions [sound, speaker icons, presented 70 ms after light **(A)** and 270 ms after light **(B)**] obtained from the FCz electrode for the TOJ and SJ tasks.

**FIGURE 10:**
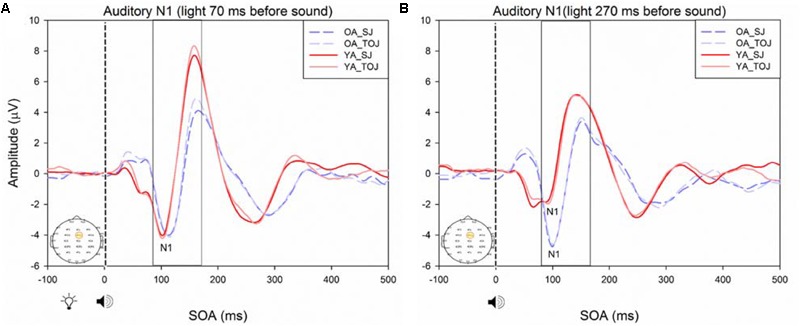
Average amplitude (μV) of auditory N1 ERPs from the experimental condition from younger and older adults in response, and time-locked, to the beep stimulus (sound icon) from the visual-auditory conditions [sound, speaker icons, presented 70 ms after light **(A)** and 270 ms after light **(B)**] obtained from the FCz electrode for the TOJ and SJ tasks.

RM ANOVAs with a 2 (group: older and younger) × 2 (task: SJ and TOJ) × 2 (SOA: 270, 70 ms) design was used to test the auditory N1 and visual P1 amplitude and latency differences between older and younger adults time-locked to sound and light. A main effect of age was found for the auditory N1 ERP from the FCz electrode when time-locked to light [*F*(1,19) = 19.51, *p* < 0.001] where older adults showed a later latency (*M* = 129.45, *SD* = 2.21) compared to younger adults (*M* = 112.96, *SD* = 2.52) (**Figure 9**). An interaction between age and SOA was found for the auditory N1 at the FCz electrode, when time-locked to sound [*F*(1,15) = 16.28, *p* = 0.001] where older adults exhibited larger auditory N1 amplitudes (*M* = -4.84, *SD* = 0.771) compared to younger adults (*M* = -3.55, *SD* = 0.39) across the SOAs whereas younger adults showed a decrease in amplitude at the 270 ms SOA (**Figure 10** and Supplementary Figure 3). In addition to auditory N1 ERP differences, visual P1 ERP differences were also found between younger and older adults. A main effect of age was found from the O2 electrode when time-locked to light [*F*(1,19) = 11.19, *p* < 0.01] where older adults showed an earlier latency (*M* = 136.54, *SD* = 3.76) compared to younger adults (*M* = 153.04, *SD* = 4.27) (Supplementary Figure 4). Additionally, a main effect of task was also found for the visual P1 ERP amplitudes when time-locked to sound from both the O2 electrode [*F*(1,19) = 6.58, *p* < 0.05] and the P4 electrode [*F*(1,17) = 9.58, *p* < 0.01] (Supplementary Figure 5). Additionally, a group difference was also found for the visual P1 ERP latency when time-locked to sound from the O2 electrode [*F*(1,19) = 11.787, *p* < 0.01] where older adults showed an earlier latency (*M* = 138.075, *SD* = 3.782) compared to younger adults (*M* = 155.075, *SD* = 3.677) (Supplementary Figure 5).

## Discussion

The present study aimed to test both within and between subject differences in younger and older adults for audiovisual simultaneity and temporal order perception. Our within-subjects behavioral results confirmed that simultaneity and temporal order perception may be subserved via different neural mechanisms. This was supported by the different TBW and PSS means obtained for the SJ and the TOJ task within younger and older adults. This was further bolstered by a lack of a significant correlation between the PSSs of the two tasks, again in both younger and older adults. Our auditory N1 and visual P1 ERP results showed that auditory and visual perception for the control conditions does not differ between the two tasks in both younger and older adults and thus indicates that performance differences found between SJ and TOJ tasks may arise from multisensory integration. Despite predicting no difference between younger and older adults for the auditory N1 ERP in the experimental condition, we found that older adults showed a sustained cortical response over the SOA of 70 ms and 270 ms compared to the N1 amplitudes obtained from younger adults indicating that older adults are integrating temporally disparate information within an extended period of time compared to younger adults. Below we discuss each of these results in more detail and in the context of other literature.

Previous work by Setti et al. (2011a) found no main effect of age between older and younger adults for the auditory N1 amplitude suggesting that auditory ERPs may be less susceptible to change compared to visual ERPs. Although we replicated Setti et al. (2011a) design, we did not find the same results, rather, our results showed that older adults exhibited larger auditory N1 amplitudes compared to younger adults across the SOAs whereas younger adults showed a decrease in amplitude at the 270 ms SOA (**Figure 9**). These results may indicate that younger adults are better able to disengage from the first stimulus in order to redirect their attention to the second stimulus whereas older adults may not as easily be able to disengage. Indeed, previous literature has shown that older adults exhibit a top-down suppression deficit of visual stimuli (Gazzaley et al., 2008). Although the behavioral results from the present study did not reach significance, they provide some support for an extended TBW in older adults in both the TOJ and SJ tasks (TOJ: *M* = 197.09, *SD* = 235.47; SJ: *M* = 263.06, *SD* = 67.26) compared to younger adults (TOJ: *M* = 157.64, *SD* = 102.03; SJ: *M* = 238.48, *SD* = 72.06). Similar to our results, the behavioral results from Setti et al. (2011a) also indicate a wider TBW in older adults compared to younger adults for the SOA of 270 ms (i.e., less accurate). However, contrary to our ERP results, Setti et al. (2011a) found smaller visual P1 and auditory N1 amplitudes in older adults compared to younger adults for the SOA of 270 ms but not for the SOA of 70 ms. Given that our project was a replication of the work conducted by Setti et al. (2011a), what could explain the differences found between these studies? One possibility is that although the older populations were similar in age (mean age = 69 years for the present study, mean age = 71 years for Setti et al. (2011a) the sampled populations may have differed in some other way. The sample of older participants utilized in our study could perhaps be healthier as they performed relatively better (i.e., narrower TBW) when compared to the sample used in Setti et al. (2011a). However, no global cognitive impairment tests were utilized in our study and thus the differences between the two groups can only be speculated. More likely, differences in the post processing of ERPs could explain this discrepancy. For example, the extraction parameters utilized in this study and in the study conducted by Setti et al. (2011a) were slightly different (i.e., for the SOA of 270 ms, time-locked to light, this study extracted the auditory N1 ERPs from 350 to 450 ms while Setti et al. (2011a) extracted from 370 to 420 ms). Other differences may have arisen from the average latency at which the maximum amplitude occurred in each study, however, we were unable to compare the differences between the two studies, as latencies were not assessed by Setti et al. (2011a). Regardless of study-specific details, there is converging evidence for an extended TBW in older adults, however, more research is required in order to determine the relationship between changes in ERPs and behavioral results.

Despite study-specific details, what is the functional relevance of our results and what can they tell us about the aging human brain? A wider TBW in older adults for both SJ and TOJ tasks indicates an impaired ability in parsing information across different modalities. Previous researchers have shown that an extended TBW is related to poor balance control (Setti et al., 2011b) and speech perception deficits (Dixon and Spitz, 1980; Hairston et al., 2005). These impairments in behavior may result from irrelevant information being integrated across the senses when the TBW is widened, leading to an erroneous perception of one’s environment. It is important to note, however, that these changes may not all be negative. Indeed, previous literature has reported that older adults show greater enhancement in performance when bimodal stimuli are presented (Diederich et al., 2008). Thus, while our results have important implications in understanding changes to the CNS that arise from aging, and could potentially inform strategies to improve older adult functions and daily living activities, much more research is required to determine the functional significance of an extended TBW in the older adult brain.

A critical factor in understanding whether changes in the older adult brain affect multisensory integration is to determine whether there are changes to unisensory processing. As was predicted, our control ERP results showed that unisensory perception of light and sound are not modulated by task in both younger and older adults. These results indicate that even when unisensory processing areas (i.e., primary auditory and visual cortices) are primed to complete a multisensory task (SJ and TOJ), the cortical activity associated with such early processing does not change over the lifespan. Our results did, however, indicate that older adults exhibited a later latency than younger adults at the O2 electrode. It should be noted that while this shift in latency was significant, we did not find a shift in PSS as would be expected. However, given the sparse number of SOAs utilized in our design, we are not confident to rule out that shifts in ERP latencies correspond with shifts in behavioral PSS. Additionally, as our study was a replication of Setti et al. (2011a), we did not include a true unisensory control condition and thus the results must be interpreted with caution. It may be that for the short SOAs (70 ms) the control conditions are not truly representing unisensory processing. As our results indicate a main effect of SOA for the visual P1 amplitude and latency from the P4 electrode, they imply that the visual modality is modulated by the auditory modality in the control condition. However, note that the purpose of the control conditions in this study was to assess whether or not there would be task differences in processing of “unimodal” information both within and between younger and older adults. Our results indicate no task differences in the control conditions, however, the differences do arise for the experimental conditions (Supplementary Figure 5) indicating that both the younger and older brain process unisensory and multimodal information differently from one another. Additionally, note that **Figure 8** is somewhat misleading as it portrays older adults as having an earlier latency. This may be caused by multiple factors including the bandpass filters we used in order to present the data more clearly. Furthermore, although both older and younger adults reported normal or corrected-to-normal vision, older adults exhibited an earlier latency than younger adults at the P4 electrode in the experimental condition. This finding conflicts with previous research where a later latency has been typically been found for visual P1 with aging (Gazzaley et al., 2008).

What could explain this result of older adults having an earlier latency than younger adults visual P1 ERP? One possible explanation may arise from shifts of attention self-generated by our participants. While we did not specifically manipulate attention in this experiment, given that the visual P1 ERP is modulated by attention (Čeponiene et al., 2008; Finnigan et al., 2011), it could be that older adults were more attentive when performing our study compared to younger adults. This at first may seem unintuitive as older adults have difficulty disengaging their attention from the first stimulus (Georgiou-Karistianis et al., 2007; Gazzaley et al., 2008) however, most older adults participating in our study reported high interest in performing well on the tasks. Hence, they may have been perceiving and processing the stimuli faster than younger adults. Finally, consistent with previous literature (Čeponiene et al., 2008), our results indicate that unisensory auditory ERPs are less susceptible to aging compared to visual ERPs, as we found no significant difference in both auditory N1 amplitude and latency between the older and younger adults in the control conditions.

Čeponiene et al. (2008) found that auditory P1 and N1 ERPs were unaffected by modulation of attention, however, the visual P1 and N1 ERPs were diminished in older adults compared to younger adults. As there is not much evidence indicating salient differences in neuro-biological aging between visual and auditory sensory regions (Spear, 1993; Sowell et al., 2003; Čeponiene et al., 2008), the differences in amplitude may *also* be explained by attention. Indeed, vision tends to be more reliant on attention than audition, as acoustic information is free to enter the auditory system regardless of attention (Čeponiene et al., 2008). Our results provide further evidence that attention may be modulating visual P1 ERPs, especially in older adults, as we failed to confirm that older adults have smaller visual P1 peak amplitudes compared to younger adults for the TOJ task.

What are the possible mechanisms that explain widening of the TBW in older adults? Multisensory integration changes over one’s lifespan, where younger adults are capable of integrating cues from multiple different senses more accurately compared to older adults (Setti et al., 2011a). Thus, determining the temporal order of events becomes exceedingly difficult with age (Setti et al., 2011a,b). These changes may be linked to many factors such as age-related changes in the concentration of the inhibitory neurotransmitter gamma-aminobutyric acid (GABA) (Takayama et al., 1992; Gao et al., 2013), or a general cognitive decline associated with age due to structural changes and loss of brain mass (Mozolic et al., 2012). It is known that decreased GABA concentration can lead to a decline in inhibitory signals, which may be associated with an inability to inhibit binding of erroneous inputs, hence leading to a loss of function (Gao et al., 2013). If general cognitive decline with aging not associated with multisensory integration could explain differences in performance between younger and older adults, performance of older adults would consistently remain poor regardless of whether unisensory or multisensory cues were presented. However, it has been found that on simple audiovisual detection tasks, reaction times (RTs) of older and younger adults are comparable for the unisensory stimuli whereas older adults had substantially faster RTs compared to younger adults on simultaneously presented audiovisual trials (Peiffer et al., 2007). Thus, age-related changes cannot fully be explained by general cognitive slowing. Multiple explanations and/or models have been proposed to explain why older adults demonstrate greater multisensory enhancement and benefit from integration of cues from different modalities. Inverse effectiveness is a potential explanation stating that when effectiveness of individual sensory stimuli is decreased, the magnitude of the multisensory integration is enhanced (Meredith and Stein, 1983, Meredith and Stein, 1986). Additionally, older adults have commonly shown wider TBWs compared to younger adults. The wider TBW may indicate that more time is available for the integration of cues from different modalities. However, Diederich et al. (2008) suggest that due to the fact that peripheral sensory processing is slower in older adults, the probability of multisensory integration is small even with a wider TBW; thus when multisensory integration does occur, the benefits are enhanced. Although our results suggest that multisensory integration is at play, this must be interpreted with caution, as other factors such as decision-making may explain the differences that arise between SJ and TOJ tasks. It has been found that the TOJ task is more complex and thus requires more resources (i.e., higher-order decision-making) compared to the SJ task (Yarrow et al., 2014), which could explain the differences seen between the two tasks and thus between younger and older adults.

Could slower unisensory processing explain our results? Although all participants were required to have normal or corrected-to-normal vision and hearing, these were assessed via self-reporting measures through both the Waterloo Research in Aging Participant Pool (WRAP) and by our lab. As self-reported measures may not be a reliable method of assessing visual and auditory acuity, it is recommended that future studies ensure that more reliable methods of testing vision and hearing are utilized to avoid such limitations. Given that sensory processing is slower in older adults, it is possible that the older adults were not integrating audiovisual cues as accurately as younger adults. As aging is associated with a higher threshold for detecting both auditory and visual cues (Gordon-Salant, 2005), our older participants may not have been perceiving the stimuli at the same intensity as younger adults, which may have affected their ability to detect the stimuli as quickly. These differences in detection may lead to slower transduction and perception of multisensory inputs in older adults (Chan et al., 2014a).

Another limitation that should be considered is the cognitive capacity of our participants. Although our results indicate that our sample of older adults performed quite well relative to the samples used in previous literature (i.e., Setti et al., 2011a) as noted earlier, the global cognition of older adults was not tested. Future researchers are strongly encouraged to utilize global cognitive tests such as the Montreal Cognitive Assessment (MoCA) or the Mini Mental State Examination (MMSE) as individual differences may contribute to the differences found between studies. Thus reporting the findings from these tests will allow for a better comparison of the types of populations researchers have utilized.

Despite some of these limitations, this work has the potential to guide future research that could include the creation and implementation of rehabilitative programs that aim at decreasing the width of the TBW in order to enhance perception of everyday events such speech comprehension and the ability to balance. Previously, researchers have exposed participants to asynchronous audiovisual stimuli in order to decrease the width of the TBW and adaptively shift the PSS (Fujisaki et al., 2004; Vroomen et al., 2004; Powers et al., 2009). More recently, Chan et al. (2014a) showed that although both younger and older adults were able to adapt to the recalibration paradigm, the magnitude of effect was smaller for older adults. We speculate that using both behavioral as well as ERP measures can help create rehabilitative programs that are able to target the TBW more effectively. Our results indicate that older adults have a wider TBW for both the SJ and the TOJ task hence recalibration of both of these tasks should be pursued. We hope this work can be used to increase the quality of life of older adults by combining both behavioral as well as neuroimaging methods to decrease the width of the TBW.

## Conclusion

Our work reveals differences in audiovisual simultaneity and temporal order perception within and between younger and older adults. Visual P1 and auditory N1 amplitudes show that unisensory perception does not differ between SJ and TOJ tasks, suggesting that multisensory integration is largely involved in driving the differences between the two tasks. Although both older and younger adults reported normal or corrected-to-normal vision and hearing, older adults exhibited later visual P1 latency in the control condition, indicating that processing of unisensory visual information may be delayed with aging. In agreement with our behavioral data indicating a wider TBW for older adults compared to younger adults, the auditory N1 ERPs implicated in multisensory integration also showed a sustained higher amplitude across the SOAs in older adults. Our results provide evidence suggesting that simultaneity and temporal order perception are processed via different neural mechanisms and that simultaneity and temporal order perception change with age.

## Author Contributions

AB and MB-C designed the study and conducted the statistical analyses. AB acquired the data. WS and MB-C critically evaluated the manuscript. All authors analyzed the data and contributed to writing the article.

## Conflict of Interest Statement

The authors declare that the research was conducted in the absence of any commercial or financial relationships that could be construed as a potential conflict of interest.
