# Supplementary material for: Simultaneity and Temporal Order Judgments Are Coded Differently and Change With Age: An Event-Related Potential Study
Source: Front Integr Neurosci. 2018 Apr 26;12:15. doi: 10.3389/fnint.2018.00015 (PMC5932149; doi:10.3389/fnint.2018.00015)
Supplement: Supplementary file 1 [file Data_Sheet_1.docx]

**SUPPLEMENTARY MATERIAL**

**Simultaneity and temporal order judgments are coded differently and change with age: an event-related potential study**

Aysha Basharat, Meaghan S Adams, W. Richard Staines, Michael-Barnett-Cowan*

*Department of Kinesiology, University of Waterloo, Waterloo, Ontario, Canada, N2L 3G1*

****: Corresponding author:* [mbc@uwaterloo.ca](mailto:mbc@uwaterloo.ca)

Pages:8, Supplementary Figures: 5


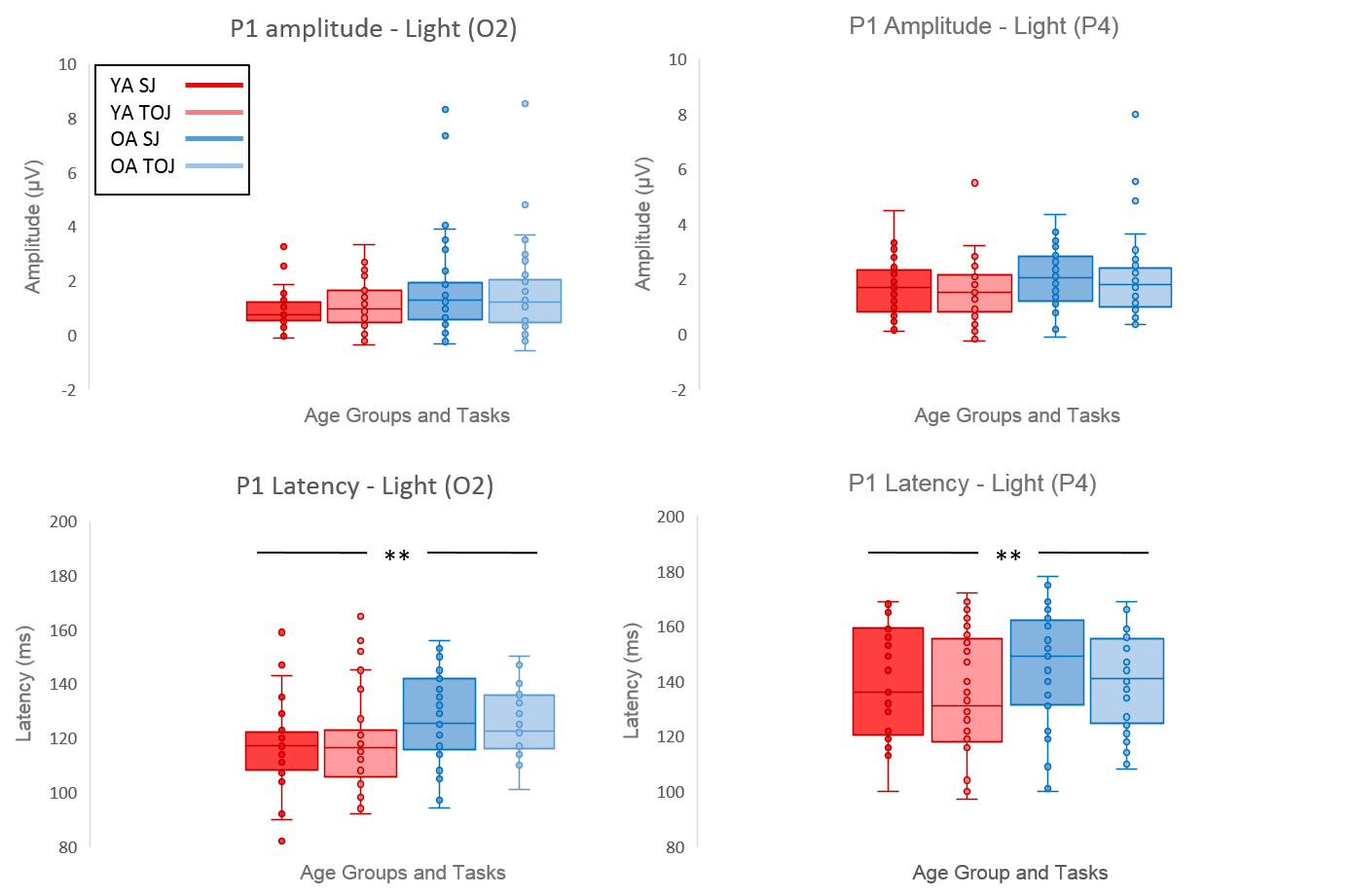


*Figure 1.* Boxplots representing the average amplitudes (µV) and latencies (ms) from younger (shades of red) and older adults (shades of blue) of the visual P1 ERPs obtained from the O2 and P4 electrodes when time-locked to light for the control condition. SJ and TOJ tasks were completed by both younger and older adults. Main effects of age were found from both the O2 and P4 electrodes where older adults showed later latencies compared to younger adults (see main text). Asterisks indicate statistical significance at the level of 0.01. The boxes have lines that extend vertically which indicate variability outside the upper and lower quartiles and any point outside those lines is considered an outlier. Note that outliers are shown here but were removed for statistical analysis.


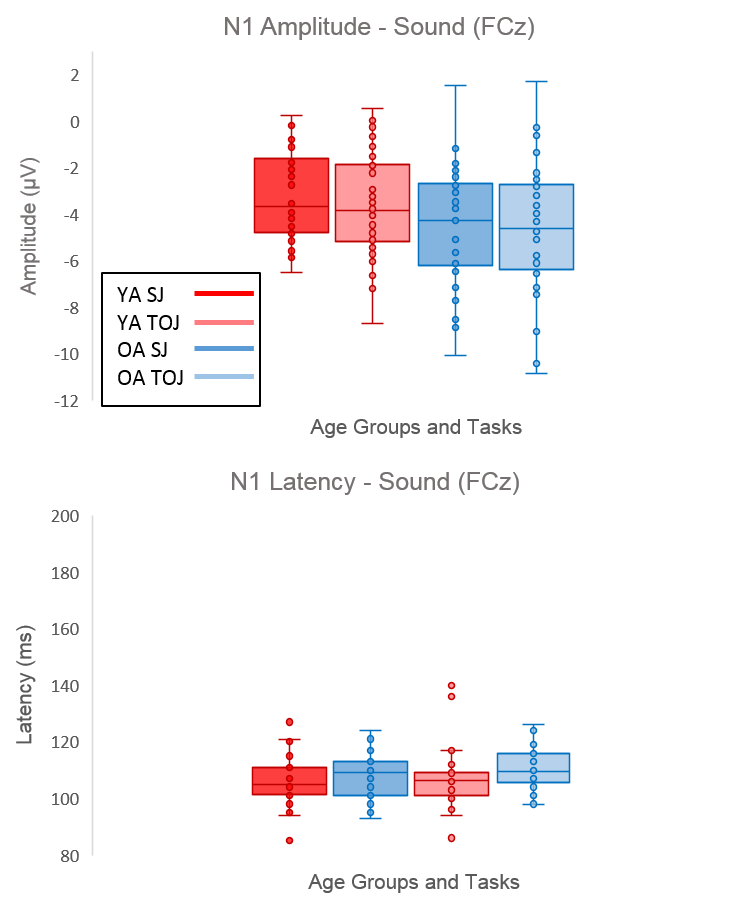


*Figure 2.* Boxplots representing the average amplitudes (µV) and latencies (ms) from younger (shades of red) and older adults (shades of blue) of the auditory N1 ERPs obtained from the FCz electrode when time-locked to sound for the control condition. SJ and TOJ tasks were completed by both younger and older adults. No statistical differences were found between younger and older adults (see main text). The boxes have lines that extend vertically which indicate variability outside the upper and lower quartiles and any point outside those lines is considered an outlier. Note that outliers are shown here but were removed for statistical analysis.


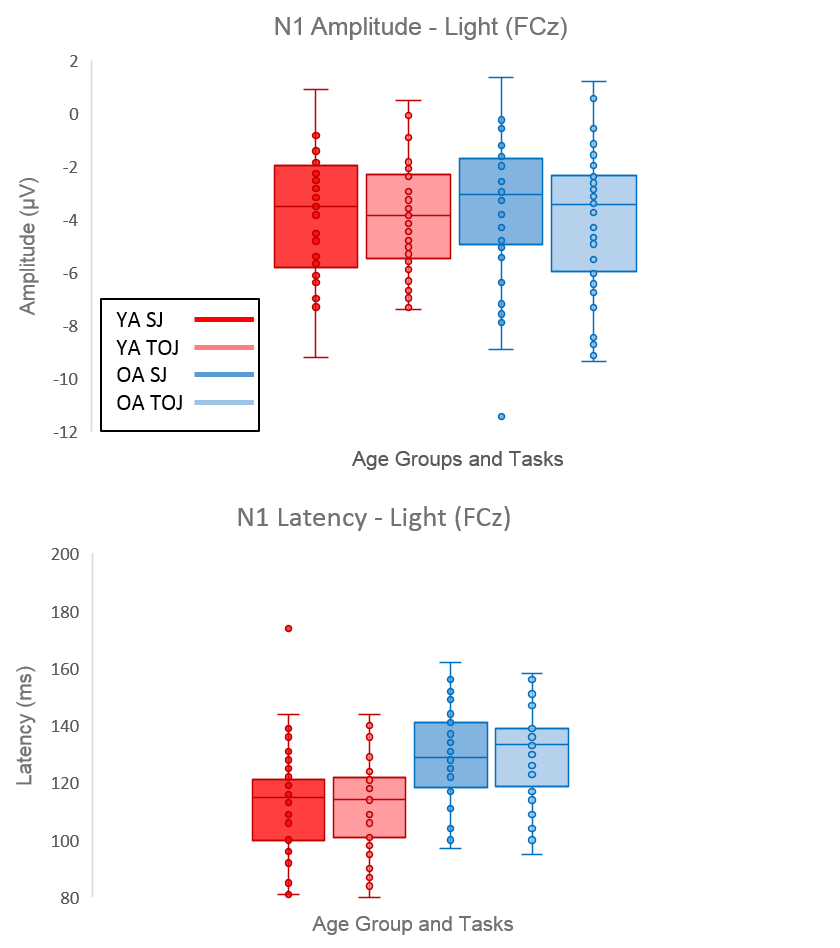


*Figure 3.* Boxplots representing the average amplitudes (µV) and latencies (ms) from younger (shades of red) and older adults (shades of blue) of the auditory N1 ERPs obtained from the FCz electrode when time-locked to light for the experimental condition. SJand TOJ tasks were completed by both younger and older adults. An interaction between age and SOA was found where older adults showed later N1 latencies compared to younger adults across the SOAs (see main text). Here, the original latencies have been modified according to SOA (subtracted by either 70 or 270 ms) in order to present the data in one figure. The boxes have lines that extend vertically which indicate variability outside the upper and lower quartiles and any point outside those lines is considered an outlier.


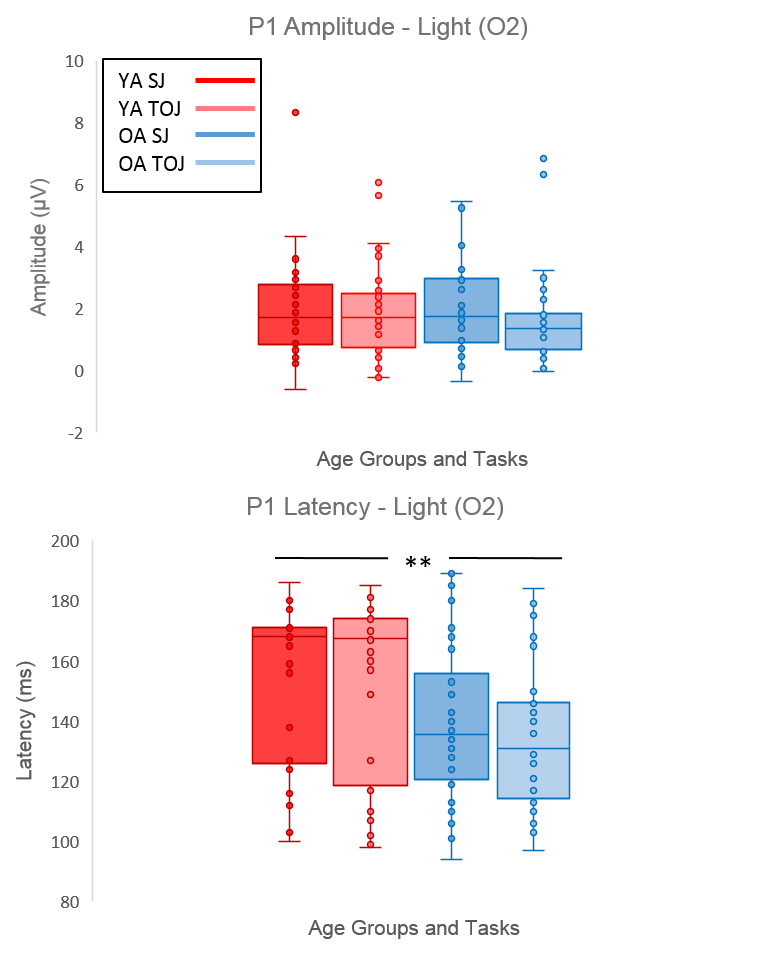


*Figure 4.* Boxplots representing the average amplitudes (µV) and latencies (ms) from younger (shades of red) and older adults (shades of blue) of the visual P1 ERPs obtained from the O2 electrode when time-locked to light for the experimental condition. SJ and TOJ tasks were completed by both younger and older adults. A main effect of age was found where older adults showed an earlier latency compared to younger adults (see main text). Asterisk indicates statistical significance at the level of 0.05. The boxes have lines that extend vertically which indicate variability outside the upper and lower quartiles and any point outside those lines is considered an outlier. Note that outliers are shown here but were removed for statistical analysis.

*
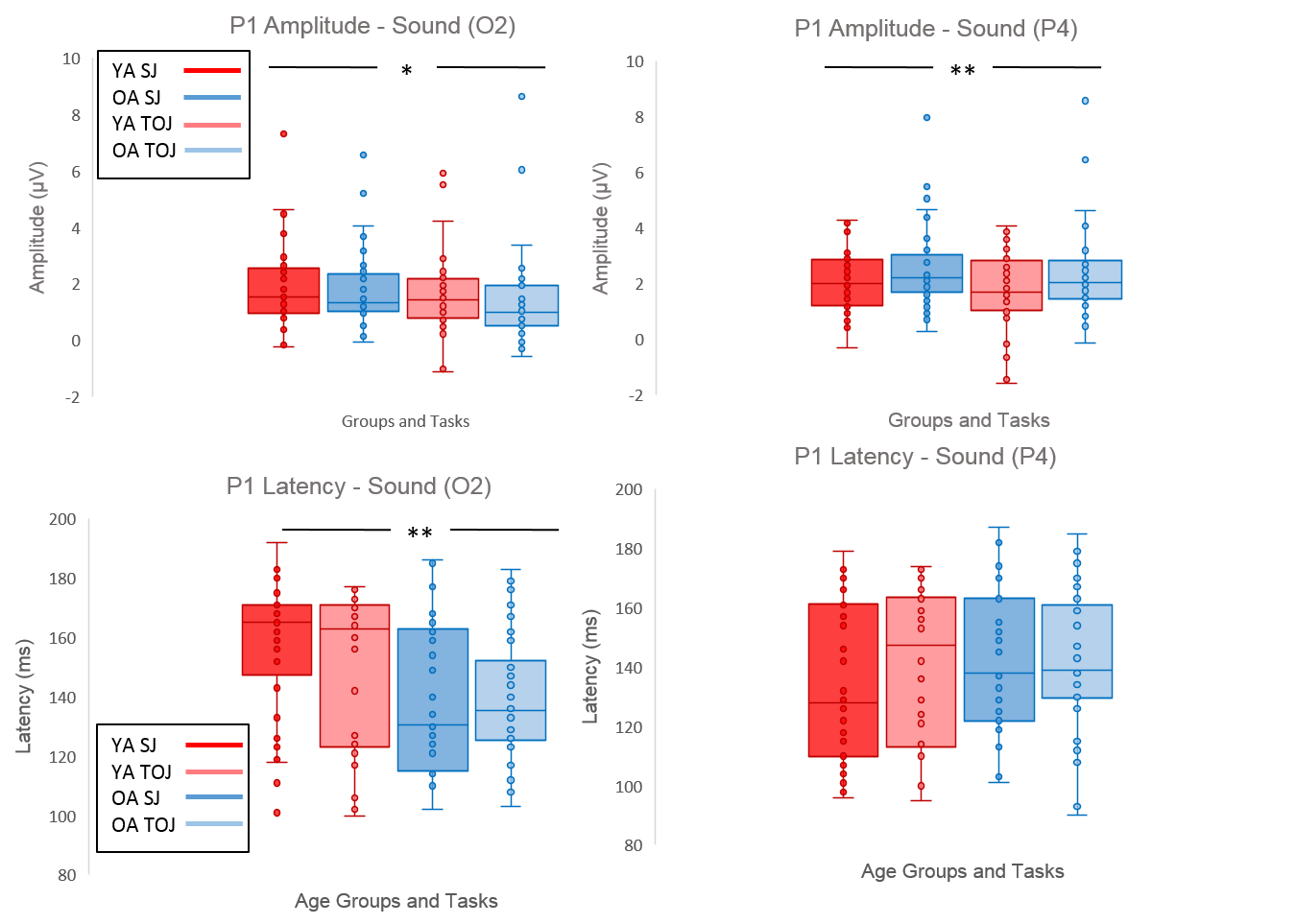
*

*Figure 5.* Boxplots representing the average amplitudes (µV) and latencies (ms) from younger (shades of red) and older adults (shades of blue) of the visual P1 ERPs obtained from the O2 and P4 electrodes when time-locked to sound for the experimental condition. SJ and TOJ tasks were completed by both younger and older adults. Main effects of task for amplitude were found where the first two boxplots are statistically different from the last two boxplots in each panel in the top row. A main effect of age was also found for latency from the O2 electrode where older adults showed a later latency than younger adults. Asterisks indicate statistical significance at the level of 0.05 and 0.01. The boxes have lines that extend vertically which indicate variability outside the upper and lower quartiles and any point outside those lines is considered an outlier. Note that outliers are shown here but were removed for statistical analysis.
